# Developmental trajectory of the corpus callosum from infancy to the juvenile stage: Comparative MRI between chimpanzees and humans

**DOI:** 10.1371/journal.pone.0179624

**Published:** 2017-06-27

**Authors:** Tomoko Sakai, Akichika Mikami, Juri Suzuki, Takako Miyabe-Nishiwaki, Mie Matsui, Masaki Tomonaga, Yuzuru Hamada, Tetsuro Matsuzawa, Hideyuki Okano, Kenichi Oishi

**Affiliations:** 1 The Russell H. Morgan Department of Radiology and Radiological Science, The Johns Hopkins University School of Medicine, Baltimore, Maryland, United States of America; 2 Department of Physiology, Keio University School of Medicine, Tokyo, Japan; 3 Primate Research Institute, Kyoto University, Inuyama, Aichi, Japan; 4 Japan Society for the Promotion of Science, Tokyo, Japan; 5 Faculty of Nursing and Rehabilitation, Chubu Gakuin University, Seki, Japan; 6 Department of Cognitive Science, Institute of Liberal Arts and Science, Kanazawa University, Ishikawa, Kanazawa, Japan; 7 Department of Psychology, Graduate School of Medicine and Pharmaceutical Science, University of Toyama, Toyama, Japan; 8 RIKEN Brain Science Institute, Laboratory for Marmoset Neural Architecture, Wako, Saitama, Japan; Instituto Cajal-CSIC, SPAIN

## Abstract

How brains develop during early life is one of the most important topics in neuroscience because it underpins the neuronal functions that mature during this period. A comparison of the neurodevelopmental patterns among humans and nonhuman primates is essential to infer evolutional changes in neuroanatomy that account for higher-order brain functions, especially those specific to humans. The corpus callosum (CC) is the major white matter bundle that connects the cerebral hemispheres, and therefore, relates to a wide variety of neuronal functions. In humans, the CC area rapidly expands during infancy, followed by relatively slow changes. In chimpanzees, based on a cross-sectional study, slow changes in the CC area during the juvenile stage and later have also been reported. However, little is known about the developmental changes during infancy. A longitudinal study is also required to validate the previous cross-sectional observations about the chimpanzee CC. The present longitudinal study of magnetic resonance imaging scans demonstrates that the CC development in chimpanzees and humans is characterized by a rapid increase during infancy, followed by gradual increase during the juvenile stage. Several differences between the two species were also identified. First, there was a tendency toward a greater increase in the CC areas during infancy in humans. Second, there was a tendency toward a greater increase in the rostrum during the juvenile stage in chimpanzees. The rostral body is known to carry fibers between the bilateral prefrontal and premotor cortices, and is involved in behavior planning and control, verbal working memory, and number conception. The rostrum is known to carry fibers between the prefrontal cortices, and is involved in attention control. The interspecies differences in the developmental trajectories of the rostral body and the rostrum might be related to evolutional changes in the brain systems.

## Introduction

The brain size of humans has increased dramatically during the evolution of *Homo sapiens*, along with the acquisition of uniquely human features, such as language, memory, self-awareness, creativity, and social communication [1–5]. Elucidating the similarities and differences in the ontogeny of brain structures between humans and our closest living primate relatives, chimpanzees, is important to understand the unique features of the human brain.

The corpus callosum (CC) is the major commissural white matter bundle that connects the left and right cerebral hemispheres and provides interhemispheric integration, which is related to sensory, motor, and higher-order cognitive functions [6, 7]. The CC is present in all primates and has evolved with the neocortex [8, 9]. The CC exhibits a topographic pattern of the different cortical areas [7, 10, 11], which is associated with different regions: the rostrum; genu; rostral body; and the anterior midbody connect regions of the prefrontal and frontal cortex; the posterior midbody connects the region of the somatosensory cortex; the isthmus connects regions of the parietal and superior temporal cortex; and the splenium connects the occipital, inferior temporal, and parietal regions [12–17]. This topographic relationship is similar in humans and chimpanzees [18].

The midsagittal area of the CC has been commonly used as a sensitive marker of brain development and maturation [19–23], since the CC area is related to number of axons and morphology, such as axon diameter and myelination [24–28]. In humans, midsagittal CC areas increase rapidly during the first two to three years of life (corresponds to infancy: note that the anthropological definition [29–36], which is different from the medical definition, is adopted in this report. See the Section “Definitions of developmental stages” for details) [37, 38] and continue to increase slowly during the juvenile stage, adolescence [21–23, 38–40], and young adulthood, until the third decade of life [19, 21, 41, 42]. In chimpanzees, a cross-sectional magnetic resonance imaging (MRI) study of the ages from 6 to 54 years (corresponds to the end of the juvenile stage to old age) indicated a gradual increase in the CC areas during this age-range, which was similar to that seen in humans. The only exception was found in the rostrum subdivision of the CC, with no significant increase during this age range [43]. Since the study did not include chimpanzees that were less than six years of age [43], whether there is a rapid increase in CC areas during infancy in chimpanzees, such as that seen in humans, is still unknown. How the rostrum develops during infancy is of particular interest, since the developmental trajectory after the juvenile stage in chimpanzees is different from that seen in humans.

To investigate the developmental changes during infancy and the juvenile stage of the chimpanzee CC, we longitudinally quantified areas of the midsagittal total CC and the CC subdivisions of four chimpanzees from 1.8 months to six years of age (infancy to the juvenile stage), using MRI, and compared the results with those of humans.

## Materials and methods

### Participants

#### Chimpanzees

Four chimpanzees (one male and three females), whose ages ranged from 1.8 to 72 months, were longitudinally evaluated during development from infancy to the juvenile stage (S1 Table). In addition, ten adult chimpanzees were cross-sectionally evaluated as references (S1 Table). Adult chimpanzee characteristics were as follows: mean (s.d.) age, 31.2 (5.8) years; female/male ratio 30 percent male. All subjects lived within a social group of 14 individuals in an enriched environment with a 700-m^2^ outdoor compound enhanced by 15-m-high climbing frames and about 500 planted trees of approximately 60 species [44], as well as an attached indoor residence at the Primate Research Institute, Kyoto University (KUPRI) [45, 46]. Access to the outdoor compound was available to all chimpanzees every other day during the day. Daily meals included a wide variety of fresh fruits and vegetables fed throughout the day, supplemented with nutritionally balanced biscuits (fed twice daily) and water available *ad libitum*. The treatment of the chimpanzees was in accordance with the 2002 and 2010 version of the Guidelines for the Care and Use of Laboratory Primates issued by KUPRI. All care and experimental protocols were approved by the Animal Welfare and Animal Care Committee of the KUPRI. Of note, one of the longitudinally-followed chimpanzees had spinal cord compression due to osteogenesis imperfecta at the T8-T11 level of the thoracic vertebrae and had paraplegia and chronic renal dysfunction. She died from pneumonia at the age of two years. The disabled infant chimpanzee was raised by her biological mother in the social group. The caretakers and veterinarians closely monitored the mother and the infant, and provided the necessary care and treatments to minimize the suffering of the chimpanzee, in accordance with the Guidelines for the Care and Use of Laboratory Primates of the KUPRI. There were neither any symptoms suggesting brain abnormality nor noticeable MRI abnormalities of the brain.

#### Humans

We used part of the previously published numerical dataset from a human cross-sectional MRI study about midsagittal CC areas. Seventy-two healthy children (40 males, 32 females), whose ages ranged from one month to 126 months (see details in [38]), were analyzed (S1 Table). The comparison with human adult CC areas was based on the data from 14 healthy adults who served as controls [38]. Adult participant characteristics were as follows: mean (s.d.) age, 19.9 (1.9) years; female/male ratio, 50 percent male (S1 Table). All parents and adult participants gave written, informed consent for participation after the nature and possible consequences of the study were explained. All the protocols of the study were approved by the Committee on Medical Ethics of Toyama University (#165).

### Image acquisition

#### Chimpanzees

Three-dimensional, T1-weighted, whole-brain images were acquired with a 0.2 Tesla MR imager (Signa Profile; General Electric). The image data from three longitudinally evaluated chimpanzees (Ayumu, Cleo, and Pal) were acquired at the following time points: 6, 12, 24, 36, 48, 60, and 72 months. Another longitudinally evaluated chimpanzee (Pico) was scanned at the following time points: 1.8, 3, 4, 6, 9, and 24 months. The chimpanzees were anesthetized with ketamine (3.5 mg/kg) and medetomidine (0.035 mg/kg), and remained anesthetized with additional ketamine (1.75 mg/kg) and/or inhalation of isoflurane or sevoflurane as needed during the scans (total time anesthetized, approximately two hours). After the scans, they were reversed with atipamezole (0.175 mg/kg) and temporarily housed in a single home cage for recovery. During the scans, the chimpanzees were placed in the scanner chamber in a supine position with their heads fitted inside either the extremity coil (for the longitudinally evaluated chimpanzees) or the head coil (for the adult chimpanzees). For the four longitudinally-evaluated chimpanzees and four of the adult chimpanzees, a three-dimensional spoiled gradient-recalled acquisition in steady state (SPGR) sequence was obtained with the following acquisition parameters: repetition time (TR), 46 ms; echo time (TE), 10 ms; flip angle, 60°; slice thickness. 1.0–2.0 mm; field of view, 20–24 cm; matrix size, 256 × 256; number of excitations, two. For the other adult chimpanzees, a three-dimensional fast gradient echo with inversion recovery prep (FGRE-IrP) sequence was obtained with the following acquisition parameters: repetition time (TR), 32.3 ms; echo time (TE), 8.5 ms; flip angle, 40°; slice thickness, 1.5 mm; field of view, 24 cm; matrix size, 256 × 256; number of excitations, two.

#### Humans

The acquisition sequence and scan procedures of the human scan have been detailed in a previous publication [38]. Briefly, three-dimensional, T1-weighted, whole human-brain images were acquired with a 1.5 Tesla MR imager (Magnetom Vision; Siemens) using the fast low angle shot three-dimensional gradient refocused (GRE) sequence. The acquisition parameters were: repetition time (TR), 35 ms; echo time (TE), 6 ms; flip angle, 35°; slice thickness, 1.5 mm; field of view, 25.6 cm; matrix size, 256 × 256; number of excitations, one.

### Image processing

#### Total CC and CC subdivisions

**Fig 1:**
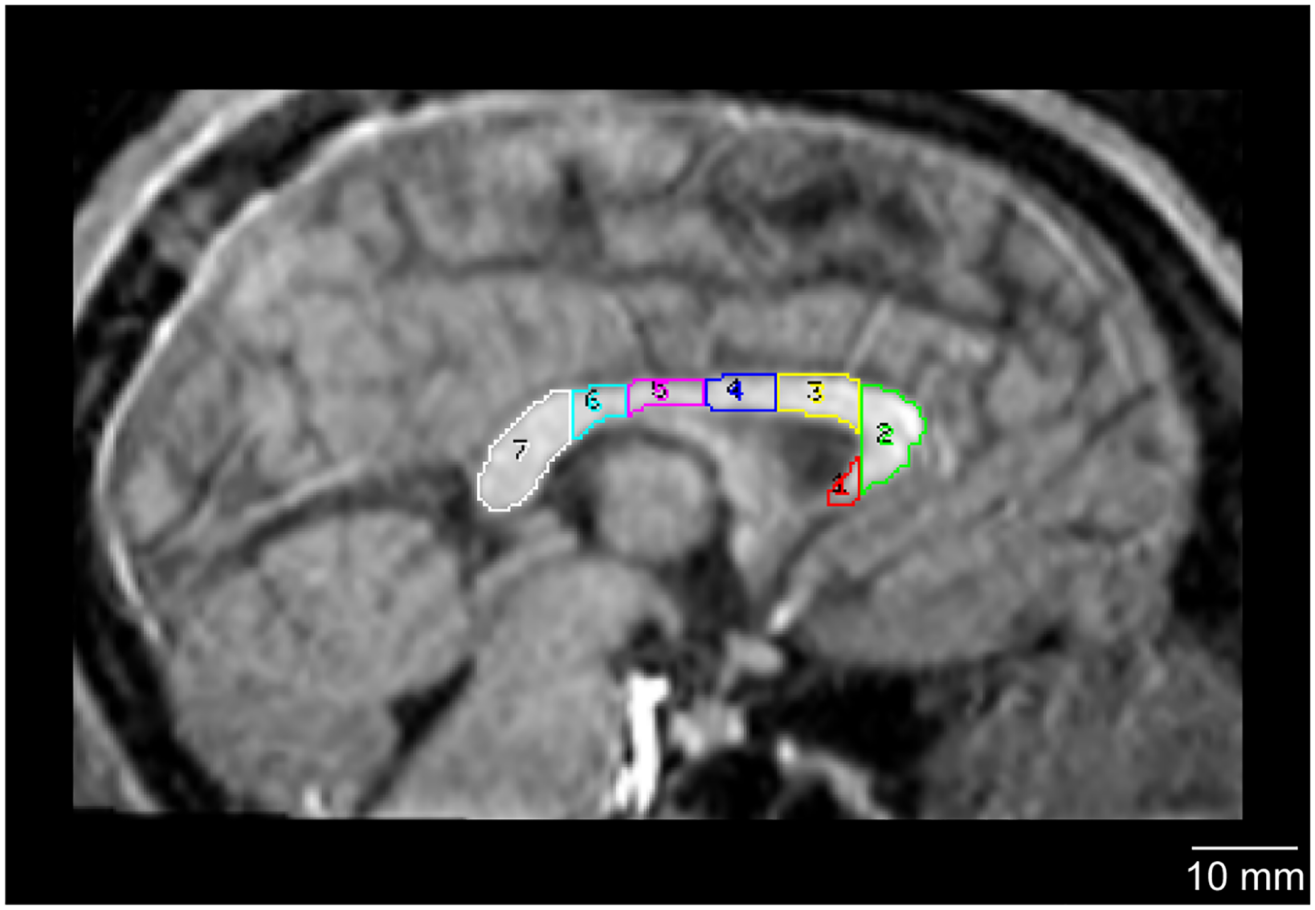
Regional subdivisions of the chimpanzee corpus callosum from a midsagittal view. The total CC midsagittal area was divided into seven equally spaced subdivisions: 1 = rostrum (red); 2 = genu (green); 3 = rostral body (yellow); 4 = anterior midbody (blue); 5 = posterior midbody (magenta); 6 = isthmus (cyan); 7 = splenium (white).

The midsagittal CC areas of the MRI for each individual were analyzed using Analyze 9.0 software (Mayo Clinic, Mayo Foundation, Rochester, MN, USA) in the following series of semi-manual procedures. (i) All images were converted into cubic voxel dimensions of 0.55 mm using a cubic spline interpolation algorithm. (ii) Brain image volumes were realigned to a standard anatomical orientation, with the transaxial plane parallel to the anterior commissure-posterior commissure line and perpendicular to the interhemispheric fissure. (iii) The midsagittal CC area measurements were obtained from the midsagittal slice in accordance with a method described by Witelson’s studies [47, 48] and previous neuroimaging studies [22, 38, 43]. (iv) This method divides the total midsagittal CC area into the subdivisions of rostrum, genu, rostral body, anterior midbody, posterior midbody, isthmus, and splenium (Fig 1). To subdivide the total midsagittal CC area, the entire length of the total midsagittal CC area was first measured, and then divided into thirds. The anterior third was further divided into three regions by tracing a vertical line through the point where the anterior CC area began to curve back slightly. This resulted in three subdivisions: the rostrum; the genu; and the rostrum body. The middle third of the overall CC area was subdivided into equal sections, resulting in the anterior midbody and posterior midbody. Finally, the posterior third of the overall CC area was subdivided into the isthmus and splenium. The splenium was defined as the posterior fifth of the entire CC area; the remaining area within the posterior third was defined as the isthmus. Using the tracing tool, the area of the CC lying within each outlined region was measured in each individual.

Two evaluators (T.S. and K.O.), who were blinded to the sex and age of the subjects, semi-manually traced and measured the midsagittal CC areas. The intra-rater and inter-rater reproducibility of the CC measurements used in this study were evaluated. Ten brain scans were randomly selected for analysis. An analysis of intra-rater reproducibility was conducted using two sets of the brain measurements obtained by T.S. The inter-rater reproducibility was analyzed by comparing brain measurements obtained by T.S. and K.O. The Pearson’s correlation coefficients for the comparisons of the results were r = 0.98 (intra-rater) and r = 0.97 (inter-rater), which indicated good reliability of the manual quantification.

#### Normalization of the total CC and the CC subdivisions relative to adult areas

To account for differences in the CC areas between adult chimpanzees and adult humans, the total CC and the CC subdivisions were normalized based on the average CC area of adult brains, and demonstrated as a percentage of adult areas (normalized area, hereafter). Since the ages of the adult chimpanzees tended to be older than that of adult humans, we investigated the effects of age on areas of total CC and the CC subdivisions in these two adult groups using a linear regression model. Total CC and the CC subdivisions served as the dependent variables. Age was introduced as an independent variable. There were no significant age-dependent changes in total CC or the CC subdivisions in either species (S2 Table). Therefore, we concluded that the adult chimpanzees and adult humans were comparable to serve as the adult control groups, given that their callosal measures were stable with respect to age.

### Definitions of developmental stages

In chimpanzees, the developmental stages were defined as follows: “infancy” corresponded to ~12 months of age, and the “juvenile stage” corresponded to ~84 months of age; in humans, these designations corresponded to ~24 months of age and ~144 months of age, respectively. The developmental stages were applied based on previous publications that used the first eruption of the first deciduous tooth [31, 32], weight increase [29, 34], and sexual maturation (menarche, first ejaculation) [30, 33, 35, 36]. The longitudinally evaluated chimpanzees of this study were observed to undergo sexual maturation at the age of seven years.

### Statistical analysis

We investigated subdivision-specific developmental trajectories in each species. For the chimpanzee study, the MRIs were obtained longitudinally from four young chimpanzees from 1.8 to 72 months of age. For the human study, MRIs were obtained from 72 children between the ages of one month and 126 months, using a cross-sectional design. These differences in study design and number of participants made the cross-species statistical comparison difficult. Therefore, we decided to remain descriptive in highlighting the similarities and differences between chimpanzees and humans, as previously reported [49–51].

#### Developmental trajectories of the total CC and the subdivisions

The relationships between the age and the CC areas were investigated by polynomial regression analyses. *F*-tests were used to determine whether the order of a developmental model was cubic, quadratic, or linear. First, linear, quadratic, or cubic polynomial regression models were fitted by age using R.v. 3.2.2 software to identify the developmental patterns in the total CC and the CC subdivisions. If a cubic model did not yield significant results, a quadratic model was tested; if a quadratic model did not yield significant results, a linear model was tested. Thus, a growth model was polynomial/nonlinear if either the cubic or quadratic term significantly contributed to the regression equation. The Akaike information (a log-likelihood function) [52] was used to ensure effective model selection.

Second, using R.v. 3.2.2 software, the data that showed nonlinear trajectories were fitted by locally weighted polynomial regression (LOESS) [53]. In this way, even with relatively few data points, the age-related area size changes could be delineated by applying the curve-fitting suggested by previous human studies [54, 55] and chimpanzee studies [49–51], without enforcing a common parametric function on the dataset, as is the case with linear polynomial models. For the fit at age X, the fit is made using values in the neighborhood of X, each weighted by the distance from X. The size of the neighborhood is defined by alpha. Data were fitted in four interactions with alpha = 0.70, in accordance with previous MRI studies [49–51, 55]. The observed and fitted values of the total CC and the CC subdivisions were plotted as a function of age to display the age-related change. The analysis was performed on the original CC areas as well as on the normalized CC areas (% of adult area, as described in the Section “Normalization of the total CC and the CC subdivisions relative to adult areas”).

#### Difference in normalized areas among CC subdivisions

We investigated the differences between the normalized areas among the CC subdivisions. This was motivated by a previous chimpanzee MRI study that indicated that there was no increase in the rostrum area in chimpanzees after the juvenile stage, while other subdivisions still developed [43]. This suggested that each subdivision has specific developmental features, and the features are potentially species-specific.

Since the study design (longitudinal vs. cross-sectional) and the number of participants (four chimpanzees vs. 72 humans) were different between the two species, we applied different statistical methods for each species. For the longitudinal dataset of the young chimpanzees, the nonparametric one-way analysis of variance (ANOVA) with repeated measures (Friedman test) (*η* ^*2*^ and *p* values) was applied to investigate within-group differences among the seven CC subdivisions. The CC subdivisions served as the test variables. When the Friedman test yielded a significant effect (p < 0.05), a post hoc analysis was performed using a Dunn's test for nonparametric pairwise comparisons between assessments, with a Bonferroni correction applied, resulting in a significance level set at *p* < 0.05.

For the cross-sectional dataset of the young humans, the parametric one-way ANOVA with repeated measures (*F* and *p* values) was applied to investigate within-group differences among the seven CC subdivisions. The CC subdivisions served as the within-subjects variables. Age was a covariate. When the parametric one-way ANOVA with repeated measures yielded a significant effect (*p* < 0.05), a post hoc analysis was performed using a paired t-test for parametric pairwise comparisons between assessments, with a Bonferroni correction applied, resulting in a significance level set at *p* < 0.05.

## Results

### Evaluation of the developmental trajectory of the total CC and the subdivisions

#### Chimpanzees

**Fig 2:**
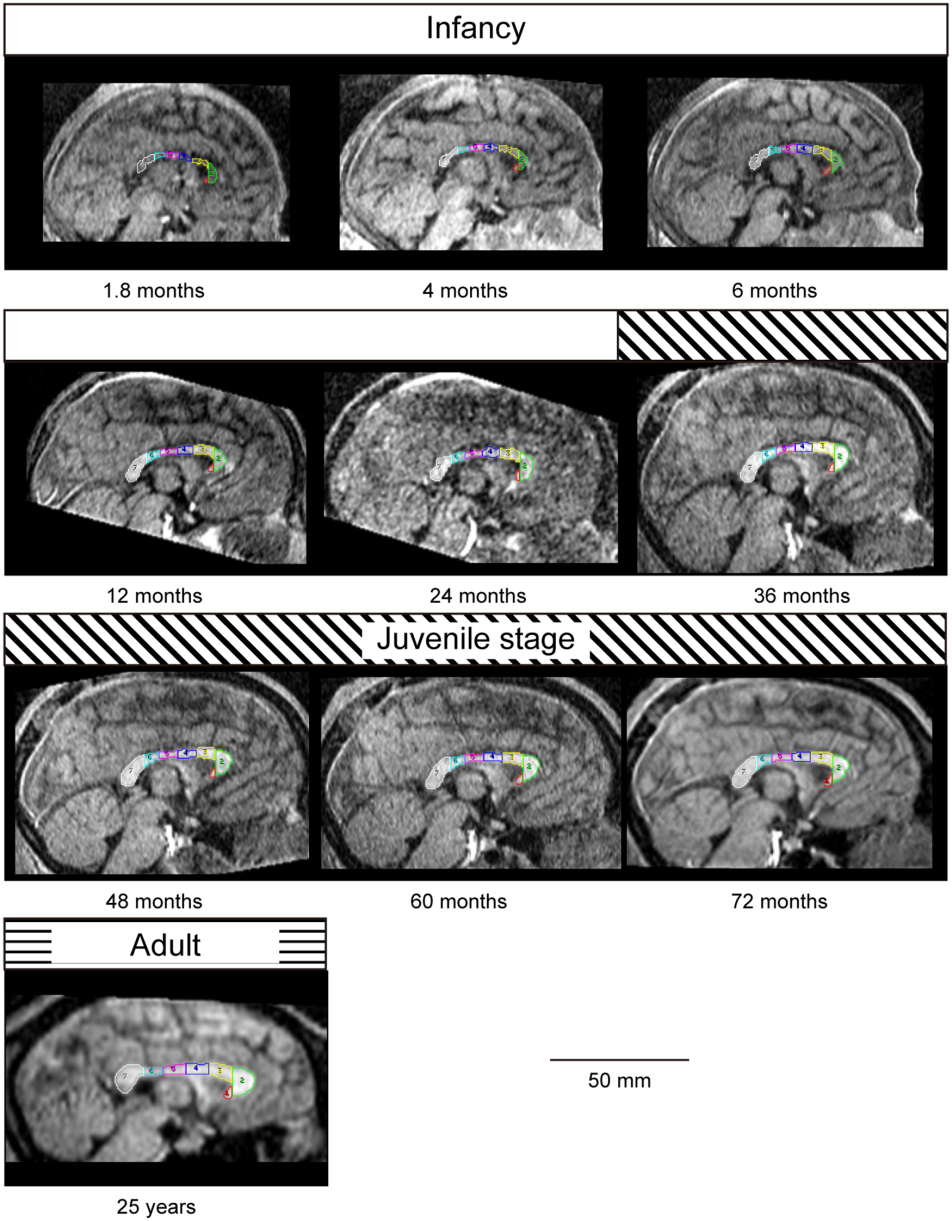
An ontogenetic series of the regional subdivisions of the chimpanzee corpus callosum from a midsagittal view. Regional subdivisions: 1 = rostrum (red); 2 = genu (green); 3 = rostral body (yellow); 4 = anterior midbody (blue); 5 = posterior midbody (magenta); 6 = isthmus (cyan); 7 = splenium (white). The bars below the figures indicate the developmental stage. The indicated developmental stages are infancy (open bar), the juvenile stage (hatched bar), and the adult stage (horizonal striped bar).

**Fig 3:**
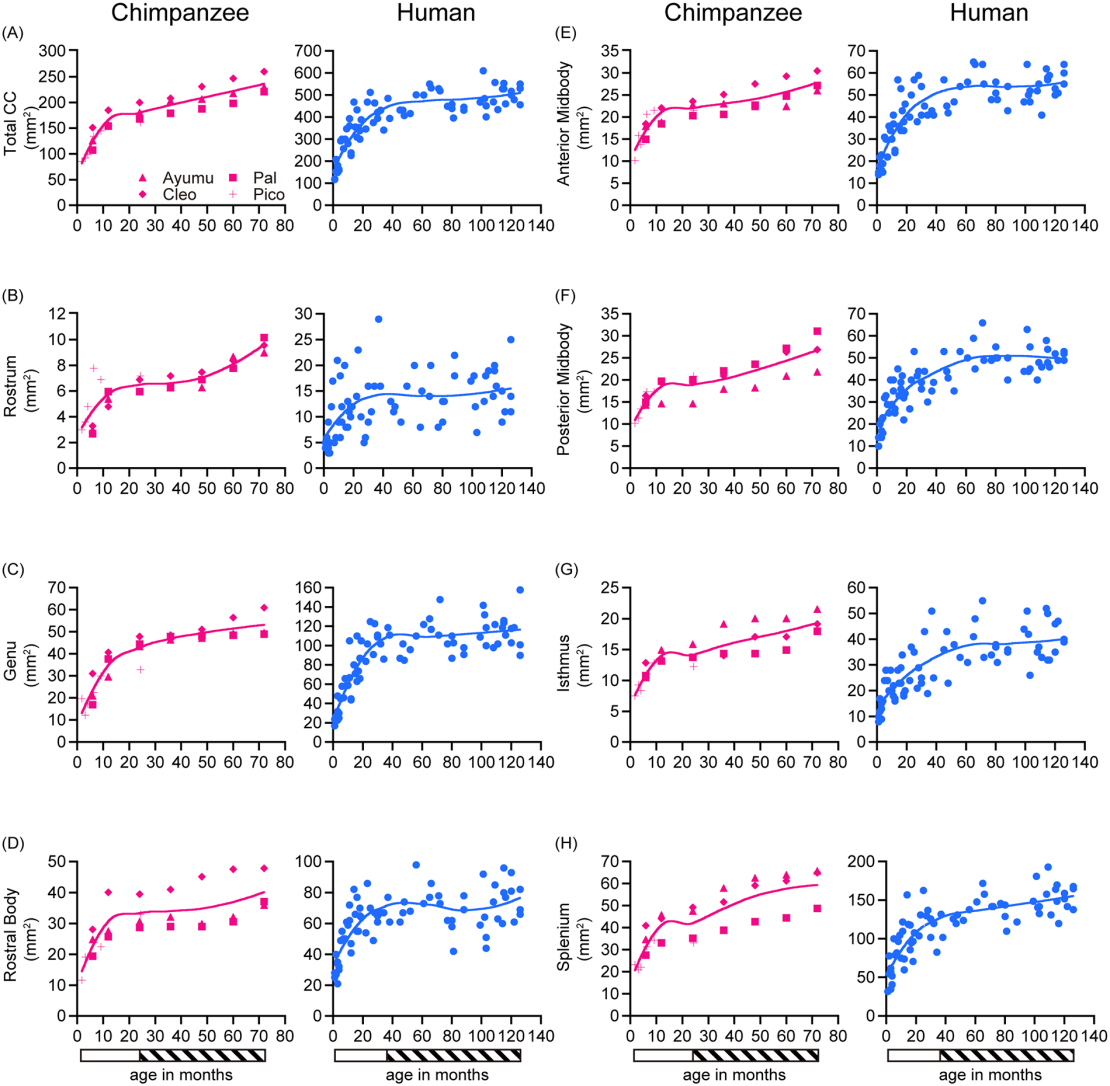
Evaluation of the corpus callosum areas during development. Age-related changes in the total CC and the CC subdivisions during infancy and the juvenile stage are shown for chimpanzees (n = 4) and humans (n = 72). (A) total, (B) rostrum, (C) genu, (D) rostral body, (E) anterior midbody, (F) posterior midbody, (G) isthmus, and (H) splenium. The bar below the graphs indicates the developmental stage. The indicated developmental stages are infancy (open bar) and the juvenile stage (hatched bar).

**Fig 4:**
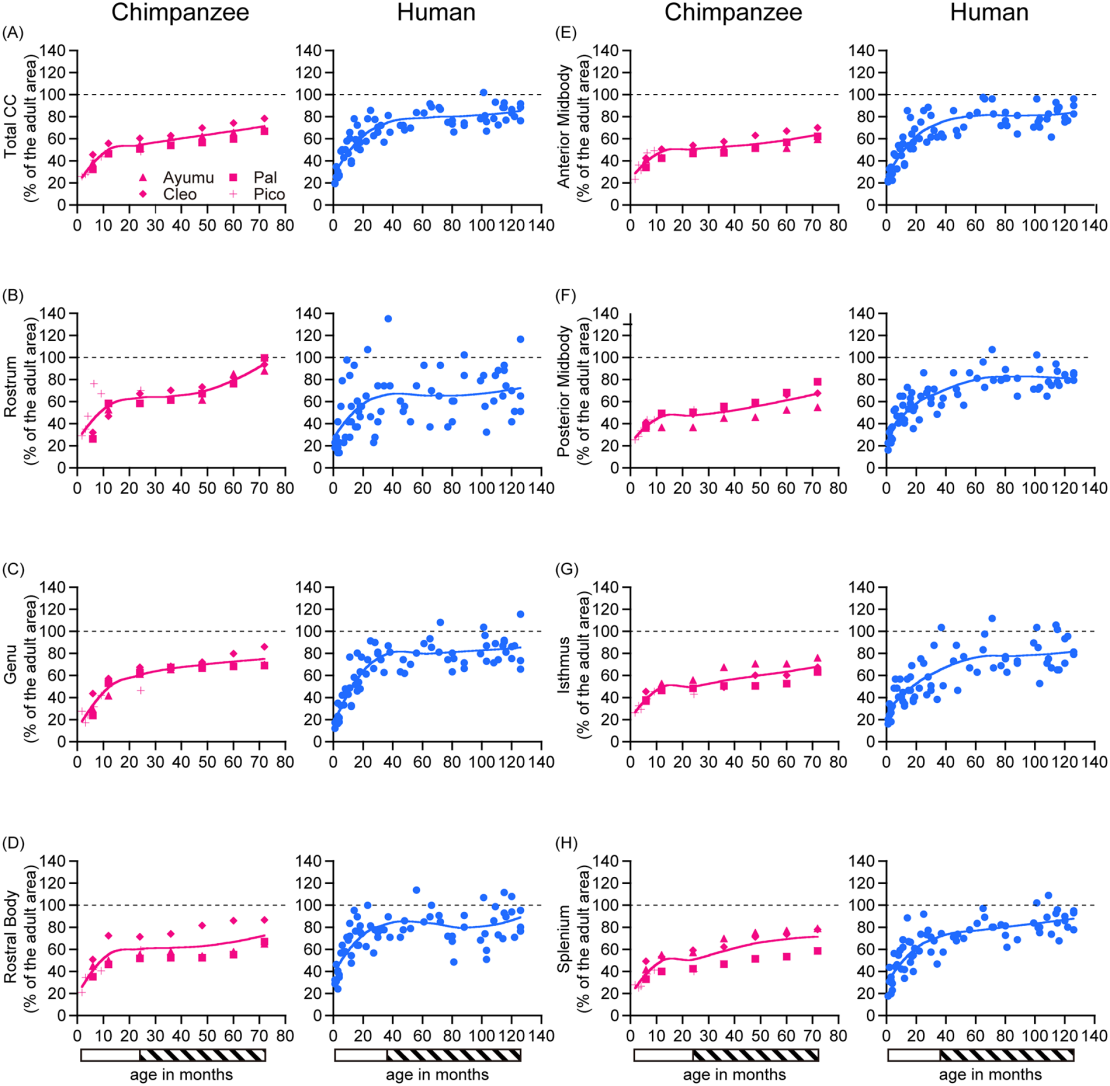
Evaluation of the normalized corpus callosum areas during development. Age-related changes in the total CC and the CC subdivisions relative to the adult areas during infancy and the juvenile stage are shown for chimpanzees (n = 4) and humans (n = 72). (A) total, (B) rostrum, (C) genu, (D) rostral body, (E) anterior midbody, (F) posterior midbody, (G) isthmus, and (H) splenium. The bar below the graphs indicates the developmental stage. The indicated developmental stages are infancy (open bar) and the juvenile stage (hatched bar).

Overall, the results of the total CC and the CC subdivisions revealed noteworthy developmental changes in chimpanzees throughout the study period (1.8 to 72 months) (Fig 2, Tables 1, 2 and 3, and S1 Table). The total CC followed a nonlinear developmental trajectory (*F* = 50.99, cubic effect, *p* = 2.55×10^−10^) (Table 3). All of the CC subdivisions also increased nonlinearly (rostrum, *F* = 23.39, cubic effect, *p* = 3.58×10^−7^; genu, *F* = 57.56, cubic effect, *p* = 7.56×10^−11^; rostral body, *F* = 11.73, cubic effect, *p* = 7.31×10^−5^; anterior midbody, *F* = 25.51, cubic effect, *p* = 1.69×10^−7^; posterior midbody, *F* = 22.02, cubic effect, *p* = 5.98×10^−7^; isthmus, *F* = 25.29, cubic effect, *p* = 1.83×10^−7^; splenium, *F* = 27.31, quadratic effect, *p* = 3.17×10^−6^) (Table 3). LOESS scatter plots for the area of the total CC and the subdivisions (in mm^2^) are demonstrated in Fig 3, and the normalized total CC and the subdivisions (in %) are demonstrated in Fig 4.

**Table 1:** Sample characteristics of the corpus callosum areas in chimpanzees.

Infants (age≤12 mons) (Number of scans = 11)		
Region	Median	IQR
Total CC	134.60	55.02
Rostrum	4.79	2.99
Genu	22.43	13.75
Rostral body	23.93	8.37
Anterior midbody	18.54	5.69
Posterior midbody	14.95	3.89
Isthmus	11.07	3.89
Splenium	33.20	17.64
Juveniles (12 mons <age≤84 mons) (Number of scans = 16)		
Region	Median	IQR
Total CC	204.72	45.23
Rostrum	7.18	2.31
Genu	48.30	2.99
Rostral body	32.45	10.54
Anterior midbody	23.33	4.49
Posterior midbody	21.39	6.06
Isthmus	16.45	5.01
Splenium	50.55	18.99

Month, mon; interquartile range, IQR. Area size characteristics of the sample classified into subgroups according to developmental stage. In chimpanzee scans (Longitudinal scan, n = 4), values represent median and IQR measured area (mm^2^).

**Table 2:** Sample characteristics of the corpus callosum areas relative to adult areas (normalized areas) in chimpanzees.

Infants (age≤12 mons) (Number of scans = 11)		
Region	Median	IQR
Total CC	40.70	16.64
Rostrum	46.97	29.32
Genu	31.70	19.43
Rostral body	43.35	15.16
Anterior midbody	42.55	13.06
Posterior midbody	37.62	9.78
Isthmus	39.13	13.74
Splenium	40.02	21.27
Juveniles (12 mons <age≤84 mons) (Number of scans = 16)		
Areas	Median	IQR
Total CC	61.90	13.67
Rostrum	70.41	22.70
Genu	68.26	4.23
Rostral body	58.78	19.08
Anterior midbody	53.54	10.30
Posterior midbody	58.14	17.69
Isthmus	60.93	22.90
Splenium	61.90	13.67

Month, mon; interquartile range, IQR. Area size characteristics of the sample classified into subgroups according to developmental stage. In chimpanzee scans (Longitudinal scan, n = 4), values represent median and IQR normalized area of the adult area (%).

**Table 3:** Results of polynomial regression modeling of the developmental trajectories of the corpus callosum areas.

Species	Region	Best fitting model	*F*	*R*^*2*^	sig
Chimpanzees	Total CC	Cubic	50.99	0.85	2.55×10^−10^
	Rostrum	Cubic	23.39	0.72	3.58×10^−7^
	Genu	Cubic	57.56	0.87	7.56×10^−11^
	Rostral body	Cubic	11.73	0.55	7.31×10^−5^
	Anterior midbody	Cubic	25.51	0.74	1.69×10^−7^
	Posterior midbody	Cubic	22.02	0.71	5.98×10^−7^
	Isthmus	Cubic	25.29	0.74	1.83×10^−7^
	Splenium	Quadratic	27.31	0.67	3.17×10^−6^
Humans	Total	Cubic	86.42	0.78	<2.20×10^−16^
	Rostrum	Cubic	7.50	0.22	2.08×10^−4^
	Genu	Cubic	73.64	0.75	<2.20×10^−16^
	Rostral body	Cubic	25.94	0.51	2.67×10^−11^
	Anterior midbody	Cubic	69.56	0.74	<2.20×10^−16^
	Posterior midbody	Cubic	59.62	0.71	<2.20×10^−16^
	Isthmus	Cubic	36.71	0.60	3.18×10^−14^
	Splenium	Cubic	52.42	0.68	<2.20×10^−16^

Age-related changes in the total CC and the CC subdivisions in chimpanzees (n = 4) and in humans (n = 72). *F* = *F* value, *R*^*2*^ = adjusted *R*^*2*^ value. “Best fitting model,” “*F*,” “*R*^*2*^,” and “sig” indicate the results of the statistical analysis of the age-related changes in the total CC and the CC subdivisions with a polynomial regression model. The best-fitting model represents the best-fitting model of the linear, quadratic, and cubic regression models.

#### Humans

As observed in chimpanzees, the results of the total CC and the CC subdivisions revealed noteworthy developmental changes in humans through the study period (one month to 126 months) (Fig 2, Tables 3, 4 and 5, and S1 Table). The total CC followed a nonlinear developmental trajectory (*F* = 86.42, cubic effect, *p* < 2.20×10^−16^) (Table 5). All of the CC subdivisions also increased nonlinearly (rostrum, *F* = 7.50, cubic effect, *p* = 2.08×10^−4^; genu, *F* = 73.64, cubic effect, *p* < 2.20×10^−16^; rostral body, *F* = 25.94, cubic effect, *p* < 2.67×10^−11^; anterior midbody, *F* = 69.56, cubic effect, *p* < 2.20×10^−16^; posterior midbody, *F* = 59.62, cubic effect, *p* < 2.20×10^−16^; isthmus, *F* = 36.71, cubic effect, *p* = 3.18×10^−14^; and splenium, *F* = 52.42, quadratic effect, *p* < 2.20×10^−16^) (Table 5). LOESS scatter plots for the area of the total CC and the subdivisions (in mm^2^) are demonstrated in Fig 3, and the normalized total CC and the subdivisions (in %) are demonstrated in Fig 4.

**Table 4:** Sample characteristics of the corpus callosum areas in humans.

Infants (age≤24 mons) (Number of scans = 36)		
Region	Mean	SD
Total CC	309.64	109.87
Rostrum	10.084	5.32
Genu	67.53	32.06
Rostral body	53.83	16.91
Anterior midbody	34.28	13.24
Posterior midbody	30.64	10.56
Isthmus	21.75	7.87
Splenium	91.53	33.98
Juveniles (24 mons <age≤144 mons) (Number of scans = 36)		
Region	Mean	SD
Total CC	481.11	92.75
Rostrum	14.75	5.08
Genu	111.7	16.95
Rostral body	71.14	13.38
Anterior midbody	53.06	10.50
Posterior midbody	48.92	8.75
Isthmus	38.17	8.08
Splenium	143.42	28.50

Month, mon; standard deviation, SD. Area size characteristics of the sample classified into subgroups according to developmental stage. In human scans (Cross-sectional scan, n = 72), values represent mean and SD normalized area of the adult area (%).

**Table 5:** Sample characteristics of the corpus callosum areas relative to adult areas (normalized areas) in humans.

Infants (age≤24 mons) (Number of scans = 36)		
Region	Mean	SD
Total CC	51.83	18.39
Rostrum	47.05	24.80
Genu	49.45	23.49
Rostral body	62.70	19.69
Anterior midbody	51.60	30.48
Posterior midbody	49.88	17.19
Isthmus	44.32	16.03
Splenium	51.82	19.24
Juveniles (24 mons <age≤144 mons) (Number of scans = 36)		
Region	Mean	SD
Total CC	80.53	8.94
Rostrum	68.83	23.70
Genu	81.76	12.41
Rostral body	82.86	15.59
Anterior midbody	79.87	10.62
Posterior midbody	79.63	11.30
Isthmus	77.78	16.46
Splenium	81.19	11.35

Month, mon; standard deviation, SD. Area size characteristics of the sample classified into subgroups according to developmental stage. In human scans (Cross-sectional scan, n = 72), values represent mean and SD measured area (mm^2^).

### Evaluation of regional variation in normalized areas among CC subdivisions

#### Chimpanzees

The nonparametric Friedman test indicated a significant main effect of the normalized subdivision areas (*η* ^*2*^ = 25.94, *P* = 2.29×10^−4^). Post hoc tests using a Dunn's test showed that the area of the rostrum differed significantly from that of the anterior midbody (*P* = 0.003), the posterior midbody (*P* = 0.002), and the isthmus (*P* = 0.007) (Table 6). The LOESS curves of the normalized CC subdivisions indicated that the increase was most rapid in the rostrum, compared to the other CC subdivisions, and the area reached close to 100% of the adult area by the end of the juvenile stage (Fig 4B–4H).

**Table 6:** Differences in normalized areas among CC subdivisions in chimpanzees.

	Rostrum	Genu	Rostral body	Anterior midbody	Posterior midbody	Isthmus	Splenium
Rostrum							
Genu	1.000						
Rostral body	1.000	1.000					
Anterior midbody	**.003**	1.000	.294				
Posterior midbody	**.002**	1.000	.247	1.000			
Isthmus	**.007**	1.000	.557	1.000	1.000		
Splenium	.064	1.000	1.000	1.000	1.000	1.000	

Values present Bonferroni-corrected *P* values. Underlined bold characters indicate a significant difference between CC subdivisions.

#### Humans

The parametric one-way repeated measures ANOVA indicated a significant main effect of subdivisions (*F* = 6.98, *P* = 4.4E-07). There was a significant interaction for age-by-subdivisions (*F* = 3.77, *P* = 0.001). Post hoc tests using a paired t-test showed that the area of the rostral body differed significantly from that of the rostrum (*P* = 7.0E-07), the genu (*P* = 0.004), the anterior midbody (*P* = 2.0E-04), the posterior midbody (*P* = 4.1E-04), the isthmus (*P* = 2.6E-06), and the splenium (*P* = 1.4E-03) (Table 7). The LOESS curves of the normalized CC subdivisions indicated that the area of the rostral body was greater than that of the other CC subdivisions at the onset of infancy, and the area reached more than 80% of the adult area by the end of infancy (Fig 4B–4H).

**Table 7:** Differences in normalized areas among CC subdivisions in humans.

	Rostrum	Genu	Rostral body	Anterior midbody	Posterior midbody	Isthmus	Splenium
Rostrum							
Genu	.286						
Rostral body	**7.0E-07**	**.004**					
Anterior midbody	.164	1.000	**2.0E-04**				
Posterior midbody	.500	1.000	**4.1E-04**	1.000			
Isthmus	1.000	.582	**2.6E-06**	.168	.560		
Splenium	.077	1.000	**1.4E-03**	1.000	1.000	.050	

Values present Bonferroni-corrected *P* values. Underlined bold characters indicate a significant difference between CC subdivisions.

### Descriptive comparison of developmental trajectories of the total CC between chimpanzees and humans

#### Similarities between chimpanzees and humans

There were two noticeable similarities. First, the total CC increased rapidly during infancy and continued to increase slowly during the juvenile stage in both species (Fig 4A). Second, the normalized total CC at the beginning of infancy was similarly small in both species. It was 25% in chimpanzees and 31% in humans (Fig 4A).

#### Differences between chimpanzees and humans

There were two noticeable differences between chimpanzees and humans. First, although the total CC increased rapidly during infancy in both species, the slope was steeper in humans than in chimpanzees. The total CC of the chimpanzees increased 220% during infancy, while the increase was 238% in humans (Fig 3A). Second, at the end of the juvenile stage, the normalized total CC of humans was greater than that of chimpanzees. In humans, the normalized area reached 85% at the end of the juvenile stage, while it remained at 71% in chimpanzees (Fig 4A).

### Descriptive comparison of developmental trajectories of the CC subdivisions between chimpanzees and humans

#### Similarities between chimpanzees and humans

There were three noticeable similarities between chimpanzees and humans. First, the proportion of each CC subdivision was similar between the two species. In adult chimpanzees, the areas of the rostrum, genu, rostral body, anterior midbody, posterior midbody, isthmus, and the splenium were 3, 21, 17, 13, 12, 9, and 25% of the total CC, respectively (S1 Table). The corresponding values for humans were 4, 23, 14, 11, 10, 8, and 30% of the total, respectively (S1 Table).

Second, the relative area of the CC subdivisions at the beginning of infancy was similar in both species, except for the rostral body (detailed in the Section “Differences between chimpanzees and humans”). In chimpanzees, the normalized CC subdivision areas at the beginning of infancy were 31% in the rostrum, 19% in the genu, 29% in the anterior midbody, 27% in the posterior midbody, 27% in the isthmus, and 25% in the splenium (Fig 4B, 4C and 4E–4H). The values in humans were 31% in the rostrum, 22% in the genu, 30% in the anterior midbody, 33% in the posterior midbody, 30% in the isthmus, and 33% in the splenium (Fig 4B, 4C and 4E–4H).

Finally, areas of the CC subdivisions increased rapidly during infancy and continued to increase slowly during the juvenile stage in both species (Fig 4C–4H). The only exception was the rostrum of chimpanzees, which is detailed in in the Section “Differences between chimpanzees and humans”.

#### Differences between chimpanzees and humans

There were four noticeable differences between the two species. First, at the beginning of infancy, the normalized rostral body area of humans was greater than that of other CC subdivisions, and, by the end of infancy, it had already reached 83% of that of the adult. This prominence of the rostral body was not seen in chimpanzees. In humans, the normalized rostral body area expands from 43% to 83% during infancy, while this expansion ranged from 26% to 60% in chimpanzees (Fig 4D).

Second, although areas of the CC subdivisions increased rapidly during infancy in both species, the slope was steeper in humans than in chimpanzees. In chimpanzees, the normalized areas of the CC subdivisions expanded during infancy from 19 to 61% in the genu, from 26 to 60% in the rostral body, from 29 to 51% in the anterior midbody, from 27 to 48% in the posterior midbody, from 27 to 50% in the isthmus, and from 25 to 51% in the splenium (Fig 4C–4H). In humans, the corresponding values were: from 22% to 80% in the genu; from 43% to 83% in the rostral body; from 30% to 74% in the anterior midbody; from 33% to 69% in the posterior midbody; from 30% to 65% in the isthmus; and from 33% to 72% in the splenium (Fig 4C–4H).

Third, the normalized areas and the CC subdivisions of humans were greater than that of chimpanzees, except for the rostrum, at the end of the juvenile stage. In chimpanzees, the normalized areas were: 75% in the genu; 73% in the rostral body; 64% in the anterior midbody; 67% in the posterior midbody; 68% in the isthmus; and 72% in the splenium at the end of juvenile stage (Fig 4C–4H). In humans, the corresponding values were: 85% in the genu; 89% in the rostral body; 84% in the anterior midbody; 81% in the posterior midbody; 82% in the isthmus; and 88% in the splenium (Fig 4C–4H).

Finally, the area of the rostrum of the chimpanzees increased more rapidly during the juvenile stage than that of the humans, and the area at the end of the juvenile stage was close to that of adults. In chimpanzees, the normalized area of the rostrum increased from 64% to 94% during juvenile stage (Fig 4B), while, in humans, the increase was from 66% to 72% (Fig 4B).

## Discussion

### General inter-species similarities and differences in the developmental trajectories

The major finding in this study was the identification, in chimpanzees, of a rapid increase in the total CC and the subdivisions during infancy, followed by a gradual increase during the juvenile stage. This developmental trajectory was similar to that reported in humans, and the increase was attributed to the formation of myelin sheaths around the axons that pass through the CC [19, 20, 24, 37, 38, 41, 56]. Accordingly, diffusion tensor imaging studies of human brains have shown that the fractional anisotropy—a measure that reflects axonal alignment, density, and myelination—increases during development [37, 41, 57–60], with a reduction in radial diffusion [61], indicating that myelination is the major cause of the volume increase.

The major differences between humans and chimpanzees were the slope of the developmental curve during infancy, which was steeper in humans than in chimpanzees, and the normalized CC areas during the juvenile stage, both of which were greater in humans than in chimpanzees (inter-species differences in the rostral body and the rostrum are discussed in the Sections “Development of the rostral body” and “Development of the rostrum”). These findings are congruent with a previous volumetric study, which indicated that cerebral volume and the substructures increased more rapidly in humans than in chimpanzees [50]. Since early infancy is critical for postnatal brain development in humans, in terms of volume increase [62, 63], synaptic elaboration, myelination [64], and the establishment of a default mode network [65], inter-species differences during this period might be related to the functional differences between humans and chimpanzees. Whether the emergence of the rapid increase during infancy is a marker of hominoids needs to be elucidated.

### Development of the rostral body

The development of the human rostral body was characterized by a greater normalized area than other CC subdivisions and a rapid increase during infancy than that of chimpanzees. This early maturation might indicate evolutional changes in brain anatomy and functions.

The rostral body carries fibers between the medial prefrontal and premotor cortices [66]. These cortical regions play an important role in behavior planning and control, such as response preparation, selection, and response control [67–69]. A DTI study of pediatric human traumatic brain injury suggested that the degree of damage in the rostral body is related to verbal working memory and mathematical concepts. A reduction in the area was also related to attention deficit hyperactivity disorder [70]. Since the functions related to the rostral body, which can be summarized as “executive functions,” are highly specialized in humans, one might argue that the early maturation seen in humans is related to cognitive and behavioral differences between humans and other hominoids. This concept must be further investigated.

### Development of the rostrum

The development of the chimpanzee rostrum was characterized by a faster increase during the juvenile stage than that of humans. The size of the area at the end of the juvenile stage was close to that of an adult. This explained why the increase in the rostrum area was not observed in the previous study that targeted chimpanzees older than our study population [43].

A histological evaluation of the primate brain showed that the rostrum carries fibers between the orbitofrontal and dorsolateral prefrontal cortices [7]. These regions are important for different processes of attention: the orbitofrontal cortex controls emotional motivation behavior, and the dorsolateral prefrontal cortex monitors cognition to develop efficient control of interfering sensory stimuli [71]. Accordingly, the rostrum is involved in the development of attention and inhibitory control [72–74], and the transfer of information between prefrontal cortices [75]. In chimpanzees, inhibitory control of saccades was weaker than that of humans, which led to frequent saccades and short fixations of eye movement [76–78]. Taken together, the development of the rostrum in chimpanzees might be related to inter-species differences in attention and inhibitory control.

### The scientific environment in chimpanzee research and the use of legacy data

In the USA, the National Institutes of Health (NIH) has ceased all invasive biomedical studies on chimpanzees [79–81]. In 2013, the NIH had decided to retire more than 300 chimpanzees, leaving 50 chimpanzees for research in case of a public-health emergency. In 2015, the NIH made the decision that they would send this remaining population to sanctuaries in subsequent years. Thus, there was a push to repurpose legacy chimpanzee brain data. After the NIH decision in 2015, a biobank of chimpanzee brains, including MRI data, was developed through NIH funding for public use (http://www.chimpanzeebrain.org/).

In Japan, our longitudinal MRI study of chimpanzee infants began in KUPRI in 2000 [49, 50], and terminated in 2012. Despite the relatively small sample size, which makes statistical analysis difficult, this longitudinally evaluated cohort is still precious and might contribute to the research community, since such a longitudinal dataset does not exist in the biobank in the USA, and it is difficult to collect new data for use now and in the future. Chimpanzees are among the endangered species and must be protected.

### Limitations

There are several limitations to our study. First, one of the chimpanzees in the present study, Pico, died at the age of two years, and had complications from spinal cord compression with paraplegia. Although there was no noticeable abnormality on the brain MRI, the existence of a subtle abnormality was difficult to detect during routine radiological evaluation. Therefore, we also analyzed the developmental trajectories without Pico’s data. We found that the results without Pico’s data did not change our conclusion obtained from the full dataset (S1 and S2 Figs; S3 Table; S1 File).

Second, in our study, the adult chimpanzees (used as references to normalize areas of the CC and the subdivisions) were scanned using two different protocols (SPGR or FGRE-IrP). To investigate the effect of scan protocol on image quantification, we compared the midsagittal CC area in a brain sample from an adult chimpanzee, scanned with SPGR and FGRE-IrP (S2 File). We found that the CC area, as measured by the two MRI sequences, are indeed close (0.2% difference) and comparable to the intra-rater difference for the manual delineation (1.7%). Therefore, we assumed that the difference in the scan protocol had little effect on the quantification of the CC area (S2 File). Since chimpanzee brain MRIs available for research purposes are limited, it is common to combine MRIs scanned with different scanners and acquisition sequences [43]. We recognize that the issue related to the heterogeneity of MRIs is one of the limitations generally seen in the field of chimpanzee neuroimaging studies.

Third, the prenatal period was not included in the present study. The inclusion of prenatal development is important since neuronal maturation at birth varies across species [82]. One possibility to enable the evaluation of prenatal development is to utilize longitudinally-acquired, legacy sonography data [51], which might make for an interesting future study.

Fourth, we defined developmental stages based on physical milestones, such as dental eruption, weight increase, and sexual maturation for the interspecies comparison, since this anthropological definition is a valid approach by which to compare the development among different primates [49, 50, 83]. However, the relationship between physical and neuronal development is not fully understood. The appropriateness of developmental staging based on neuronal milestones has yet to be investigated.

Finally, the method used to draw the boundaries of the seven CC subdivisions on T1-weighted MRI was based on the studies about the topographical organization of the white matter fibers of the adult human brain. The applicability of the MRI-based anatomical definition throughout different developmental stages has not been fully validated. Cross-species longitudinal evaluation of the topological organization of CC fibers during development is essential for validation.

In summary, our results suggest that CC development in chimpanzees and humans is characterized by a rapid increase during infancy, followed by a relatively slow increase during the juvenile stage. The differences between the two species include a tendency toward a greater increase in the human CC areas, especially in the rostral body, during infancy, compared to that observed in chimpanzees. A tendency toward a greater increase in the rostrum during the juvenile stage in chimpanzees, compared to that observed in humans, was also observed. The interspecies differences in the developmental trajectories of the rostral body and the rostrum might underpin evolutional changes in the executive functions related to these areas.

## Supporting information

S1 Fig Evaluation of the rostrum and genu during development in young chimpanzee data with and without Pico’s data. Age-related changes in the rostrum and genu during infancy and the juvenile stage (6 to 72 months) are shown for chimpanzees with and without Pico’s data (n = 4; n = 3). (A) rostrum, (B) genu. The bar below the graphs indicates the developmental stage. The indicated developmental stages are infancy (open bar) and the juvenile stage (hatched bar).
(PDF)

S2 Fig Evaluation of the normalized rostrum and genu during development in chimpanzee data with and without Pico’s data. Age-related changes in the rostrum and genu, relative to the adult areas, during infancy and the juvenile stage (6 to 72 months) are shown for chimpanzees with and without Pico’s data (n = 4; n = 3). (A) rostrum, (B) genu. The bar below the graphs indicates the developmental stage. The indicated developmental stages are infancy (open bar) and the juvenile stage (hatched bar).
(PDF)

S1 Table Age, total CC, and CC subdivisions in chimpanzees and humans during the developmental course of the study period. (PDF)

S2 Table Results of the linear regression model of age-related changes in the corpus callosum areas during the adult stage. Age-related changes in the total CC and the CC subdivisions during the adult stage (n = 10; mean (s.d.) age, 31.2 (5.8) years). *F* = *F* value, *R*^*2*^ = adjusted *R*^*2*^ value. “*F*,” “*R*^*2*^,” and “sig” indicate the results of the statistical analysis for the age-related changes in the total CC and the CC subdivisions with a linear regression model. “n.s.” indicates “not significant.”
(DOCX)

S3 Table Results of polynomial regression modeling of the developmental trajectories of the rostrum and genu in chimpanzees with and without Pico’s data. Age-related changes in the rostrum and genu in chimpanzees with and without Pico’s data (n = 4; n = 3). *F* = *F* value, *R*^*2*^ = adjusted *R*^*2*^ value. “Best fitting model,” “*F*” “*R*^*2*^,” and “sig” indicate the results of the statistical analysis for the age-related changes in the rostrum and genu with a polynomial regression model. The best-fitting model represents the best-fitting model of the linear, quadratic, and cubic regression models.
(DOCX)

S1 File Comparison of the developmental trajectories of the rostrum and genu in young chimpanzees with and without Pico’s data. (DOCX)

S2 File Comparison of the measurements of the chimpanzee CC area acquired with different MRI sequences. (DOCX)
