# Supplementary figures and images for: Developmental trajectory of the corpus callosum from infancy to the juvenile stage: Comparative MRI between chimpanzees and humans
Source: PLoS One. 2017 Jun 27;12(6):e0179624. doi: 10.1371/journal.pone.0179624 (PMC5487015; doi:10.1371/journal.pone.0179624)

## Chimpanzees

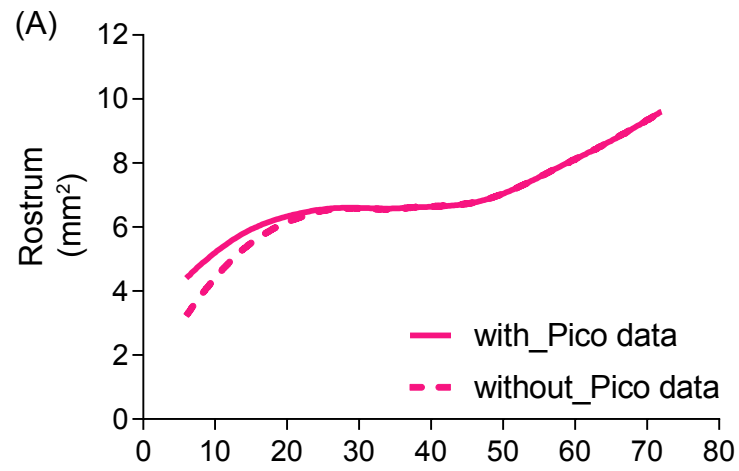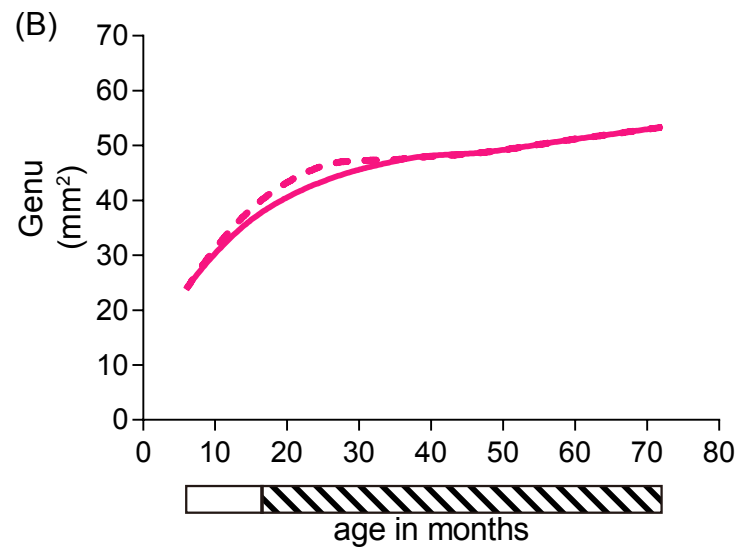

Supplement: S1 Fig — Age-related changes in the rostrum and genu during infancy and the juvenile stage (6 to 72 months) are shown for chimpanzees with and without Pico’s data (n = 4; n = 3). (A) rostrum, (B) genu. The bar below the graphs indicates the developmental stage. The indicated developmental stages are infancy (open bar) and the juvenile stage (hatched bar). (PDF) [file pone.0179624.s001.pdf]

## Chimpanzees

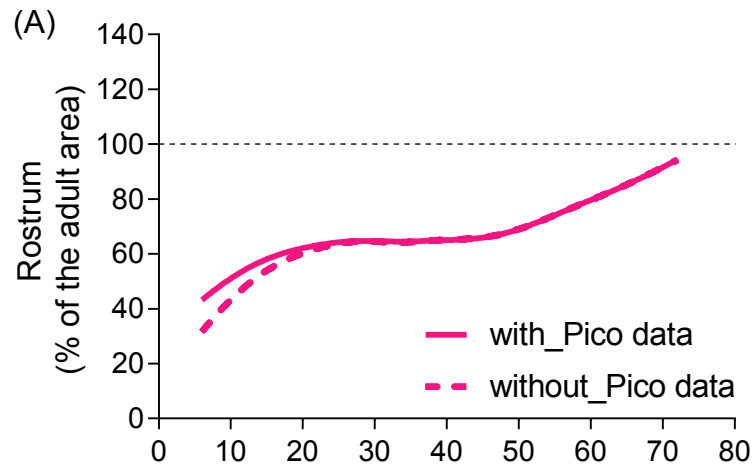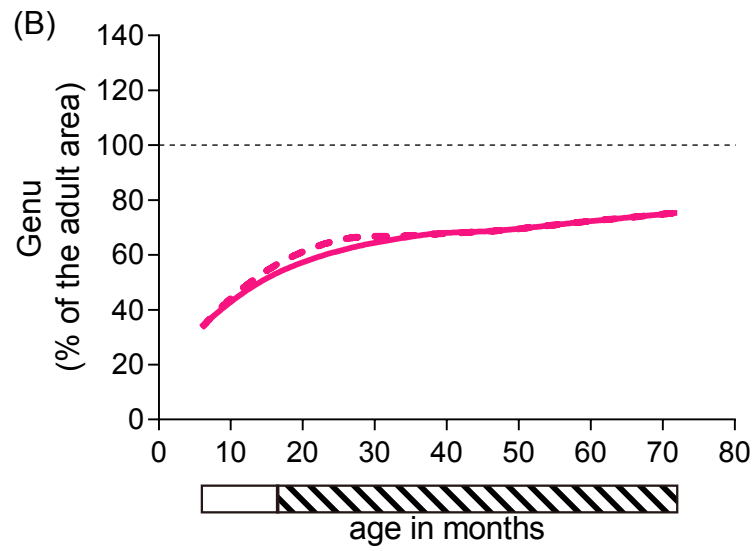

Supplement: S2 Fig — Age-related changes in the rostrum and genu, relative to the adult areas, during infancy and the juvenile stage (6 to 72 months) are shown for chimpanzees with and without Pico’s data (n = 4; n = 3). (A) rostrum, (B) genu. The bar below the graphs indicates the developmental stage. The indicated developmental stages are infancy (open bar) and the juvenile stage (hatched bar). (PDF) [file pone.0179624.s002.pdf]
