# Supplementary material for: Developmental trajectory of the corpus callosum from infancy to the juvenile stage: Comparative MRI between chimpanzees and humans
Source: PLoS One. 2017 Jun 27;12(6):e0179624. doi: 10.1371/journal.pone.0179624 (PMC5487015; doi:10.1371/journal.pone.0179624)
Supplement: S1 Table — (PDF) [file pone.0179624.s003.pdf]

Table S1. Age, total CC and CC subdivisions in chimpanzees and humans during the developmental course of the study period.

				CC area (mm^2)							
Subject	Species	Sex	Age (months)	Rostrum	Genu	Rostral Body	Anterior Midbody	Posterior Midbody	Isthmus	Splenium	Total
Ayumu	Chimp	M	6	2.99	20.94	24.82	17.94	14.36	10.47	34.69	126.21
Ayumu	Chimp	M	12	5.38	29.61	27.51	18.84	14.65	14.95	45.76	156.70
Ayumu	Chimp	M	24	5.98	43.37	30.51	22.73	14.65	15.85	47.55	180.64
Ayumu	Chimp	M	36	6.28	46.36	32.00	23.03	17.94	19.14	58.02	202.77
Ayumu	Chimp	M	48	6.28	47.25	29.91	22.43	18.24	20.04	62.51	206.66
Ayumu	Chimp	M	60	8.67	49.35	32.00	22.43	20.93	20.04	64.00	217.42
Ayumu	Chimp	M	72	8.97	49.65	35.89	26.02	21.83	21.53	65.80	229.69
Cleo	Chimp	F	6	3.29	31.10	28.11	18.54	16.45	12.86	40.97	151.32
Cleo	Chimp	F	12	4.79	40.67	40.08	22.13	19.44	13.46	44.26	184.83
Cleo	Chimp	F	24	6.88	47.85	39.48	23.63	19.44	13.76	49.35	200.39
Cleo	Chimp	F	36	7.18	48.45	40.97	25.12	20.94	14.06	51.74	208.46
Cleo	Chimp	F	48	7.48	51.14	45.16	27.51	23.63	17.05	59.22	231.19
Cleo	Chimp	F	60	8.37	56.52	47.55	29.31	26.32	17.05	61.31	246.43
Cleo	Chimp	F	72	9.57	61.01	47.85	30.51	26.92	19.14	64.90	259.90
Pal	Chimp	F	6	2.69	17.05	19.44	14.95	14.95	10.77	27.51	107.36
Pal	Chimp	F	12	5.98	37.68	25.72	18.54	19.74	13.16	33.20	154.02
Pal	Chimp	F	24	5.98	44.56	28.71	20.34	20.04	13.76	35.29	168.68
Pal	Chimp	F	36	6.28	47.85	29.01	20.64	22.13	14.36	38.88	179.15
Pal	Chimp	F	48	6.88	48.15	29.01	22.73	23.63	14.36	42.77	187.53
Pal	Chimp	F	60	7.78	48.45	30.51	24.82	27.22	14.95	44.56	198.29
Pal	Chimp	F	72	10.17	49.05	37.08	27.22	31.10	17.94	48.75	221.31
Pico	Chimp	F	1.8	2.99	19.74	11.66	10.17	10.17	7.48	23.33	85.54
Pico	Chimp	F	3	3.59	12.26	19.14	15.85	11.36	9.27	20.94	92.41
Pico	Chimp	F	4	4.79	17.35	19.14	13.76	13.46	8.37	22.13	99.00
Pico	Chimp	F	6	7.78	22.43	23.93	20.64	17.35	11.07	31.40	134.60
Pico	Chimp	F	9	6.88	29.91	22.43	21.53	17.35	12.86	34.39	145.35
Pico	Chimp	F	24	7.18	32.90	32.90	21.53	20.93	12.26	33.20	160.90
Akira	Chimp	M	388	5.38	42.47	51.44	34.99	28.11	26.02	72.67	261.08
Gon	Chimp	M	435	8.97	72.08	66.09	47.85	39.48	25.72	96.30	356.49
Reo	Chimp	M	278	11.07	86.13	67.59	49.35	34.69	20.64	87.93	357.40
Ai	Chimp	F	382	26.32	100.79	66.09	48.75	46.95	40.67	105.87	435.44
Cloe	Chimp	F	363	5.98	76.54	38.57	53.52	47.54	33.79	72.06	328.00
Mari	Chimp	F	393	10.17	67.59	73.57	50.54	49.65	39.18	102.58	393.28
Pan	Chimp	F	312	5.38	62.81	36.79	35.59	31.10	22.13	65.80	259.60
Popo	Chimp	F	294	11.96	77.46	46.06	36.79	30.80	22.43	79.85	305.35
Puchi	Chimp	F	385	10.47	46.36	52.04	31.70	37.38	22.43	60.71	261.09
Reiko	Chimp	F	512	6.28	75.37	53.83	46.66	51.74	29.91	85.83	349.62
1	Human	M	1	5	17	28	14	14	10	32	120
2	Human	M	1	4	24	25	15	10	8	32	118
3	Human	M	2	5	27	40	22	20	17	78	209
4	Human	M	3	9	23	34	16	17	15	35	149
5	Human	M	3	4	32	35	23	22	9	57	182
6	Human	M	4	3	25	32	19	23	16	41	159
7	Human	M	5	12	45	49	31	32	24	100	293
8	Human	M	7	5	46	50	22	29	24	82	258
9	Human	M	8	6	66	50	36	25	20	97	300
10	Human	M	9	21	59	61	34	40	28	105	348
11	Human	M	10	9	59	59	30	29	20	75	281
12	Human	M	11	18	67	72	41	35	18	110	361
13	Human	M	12	12	45	41	24	27	16	74	239
14	Human	M	12	9	44	47	25	25	17	60	227
15	Human	M	14	20	80	82	57	45	28	157	469
16	Human	M	15	8	71	66	36	33	23	119	356
17	Human	M	16	8	110	77	53	39	29	118	434
18	Human	M	19	12	105	64	46	34	20	108	389
19	Human	M	23	23	102	86	50	40	37	131	469
20	Human	M	25	10	125	70	57	53	35	163	513
21	Human	M	27	5	91	49	38	42	21	130	376
22	Human	M	28	6	123	67	59	44	29	137	465
23	Human	M	30	9	109	65	54	36	23	126	422
24	Human	M	34	16	86	67	41	30	19	83	342
25	Human	M	37	29	119	77	43	35	51	132	486

26	Human	M	45	13	87	61	57	53	36	126	433
27	Human	M	52	9	96	68	47	35	38	124	417
28	Human	M	56	16	110	98	54	53	33	138	502
29	Human	M	65	8	128	77	65	59	41	172	550
30	Human	M	66	15	117	86	64	45	48	158	533
31	Human	M	80	9	110	58	51	50	37	144	459
32	Human	M	81	13	103	42	48	50	31	110	397
33	Human	M	88	22	91	78	43	40	36	122	432
34	Human	M	88	18	98	69	54	50	35	122	446
35	Human	M	99	17	109	59	47	44	44	149	469
36	Human	M	101	18	142	92	64	63	51	181	611
37	Human	M	116	16	111	77	59	46	32	120	461
38	Human	M	126	25	101	82	64	52	39	168	531
39	Human	M	126	11	90	66	60	53	39	138	457
40	Human	M	126	14	158	69	55	49	40	165	550
41	Human	F	2	6	22	27	18	14	12	55	154
42	Human	F	3	3	48	21	16	14	13	62	177
43	Human	F	4	5	30	30	15	16	16	52	164
44	Human	F	6	17	58	55	30	33	28	79	300
45	Human	F	11	6	105	62	37	40	22	122	394
46	Human	F	16	13	63	55	34	36	23	86	310
47	Human	F	17	11	74	55	43	42	34	95	354
48	Human	F	18	12	87	65	38	22	19	97	340
49	Human	F	18	10	66	70	34	32	18	71	301
50	Human	F	22	14	84	54	40	27	24	104	347
51	Human	F	30	16	111	61	41	39	25	102	395
52	Human	F	32	11	102	67	45	44	43	120	432
53	Human	F	38	16	111	61	41	39	25	102	395
54	Human	F	47	11	102	67	45	44	43	120	432
55	Human	F	48	12	85	74	42	41	23	131	408
56	Human	F	61	20	122	73	56	50	31	148	500
57	Human	F	71	14	111	78	64	66	55	142	530
58	Human	F	72	20	148	73	55	49	33	142	520
59	Human	F	77	8	102	62	48	49	34	146	449
60	Human	F	80	13	87	62	56	55	38	129	440
61	Human	F	102	12	132	64	50	44	42	141	485
62	Human	F	103	7	100	44	47	45	26	133	402
63	Human	F	103	7	119	51	52	55	26	142	452
64	Human	F	108	13	98	74	52	40	35	158	470
65	Human	F	109	18	122	85	55	48	37	193	558
66	Human	F	111	19	102	63	41	46	33	131	435
67	Human	F	114	14	119	60	62	58	52	164	529
68	Human	F	115	19	126	96	57	54	32	170	554
69	Human	F	115	20	124	80	58	48	50	149	529
70	Human	F	120	9	118	81	53	52	35	152	500
71	Human	F	120	15	103	93	50	52	46	159	518
72	Human	F	122	11	119	61	51	49	47	142	480
114	Human	M	216	15	171	78	76	85	61	197	683
143	Human	M	223	32	156	91	88	77	53	172	669
173	Human	M	238	41	187	73	53	41	56	177	628
174	Human	M	244	17	138	71	67	58	60	185	596
183	Human	M	244	19	146	94	65	56	40	173	593
175	Human	M	245	11	155	82	72	71	65	163	619
172	Human	M	301	38	110	96	66	48	32	161	551
052	Human	F	216	21	90	83	61	54	37	158	504
141	Human	F	224	19	131	90	75	72	47	200	634
171	Human	F	238	10	114	69	52	62	43	157	507
134	Human	F	244	24	146	102	72	66	50	178	638
168	Human	F	248	9	132	86	67	55	39	191	579
169	Human	F	253	23	126	95	59	61	46	206	616
170	Human	F	268	21	110	92	57	54	58	155	547
