# Supplementary material for: Developmental trajectory of the corpus callosum from infancy to the juvenile stage: Comparative MRI between chimpanzees and humans
Source: PLoS One. 2017 Jun 27;12(6):e0179624. doi: 10.1371/journal.pone.0179624 (PMC5487015; doi:10.1371/journal.pone.0179624)
Supplement: S2 Table — Age-related changes in the total CC and the CC subdivisions during the adult stage (n = 10; mean (s.d.) age, 31.2 (5.8) years). F = F value, R2 = adjusted R2 value. “F,” “R2,” and “sig” indicate the results of the statistical analysis for the age-related changes in the total CC and the CC subdivisions with a linear regression model. “n.s.” indicates “not significant.” (DOCX) [file pone.0179624.s004.docx]

**S2 Table. Results of linear regression modeling of the age-related changes in the corpus callosum areas during the adult stage.**

|  | Region | *F* | *R^2^* | sig |
| --- | --- | --- | --- | --- |
| Chimpanzees | Total CC | 0.428 | n.s. | 0.531 |
|  | Rostrum | 0.101 | n.s. | 0.758 |
|  | Genu | 0.127 | n.s. | 0.731 |
|  | Rostral body | 0.382 | n.s. | 0.554 |
|  | Anterior midbody | 0.334 | n.s. | 0.579 |
|  | Posterior midbody | 5.001 | n.s. | 0.056 |
|  | Isthmus | 1.482 | n.s. | 0.258 |
|  |  |  |  |  |
|  |  |  |  |  |
| Humans | Total CC | 1.582 | n.s. | 0.232 |
|  | Rostrum | 1.223 | n.s. | 0.290 |
|  | Genu | 1.320 | n.s. | 0.273 |
|  | Rostral body | 1.925 | n.s. | 0.190 |
|  | Anterior midbody | 1.290 | n.s. | 0.278 |
|  | Posterior midbody | 4.915 | n.s. | 0.047 |
|  | Isthmus | 1.123 | n.s. | 0.310 |
|  | Splenium | 1.072 | n.s. | 0.321 |
