# Supplementary material for: Developmental trajectory of the corpus callosum from infancy to the juvenile stage: Comparative MRI between chimpanzees and humans
Source: PLoS One. 2017 Jun 27;12(6):e0179624. doi: 10.1371/journal.pone.0179624 (PMC5487015; doi:10.1371/journal.pone.0179624)
Supplement: S3 Table — Age-related changes in the rostrum and genu in chimpanzees with and without Pico’s data (n = 4; n = 3). F = F value, R2 = adjusted R2 value. “Best fitting model,” “F” “R2,” and “sig” indicate the results of the statistical analysis for the age-related changes in the rostrum and genu with a polynomial regression model. The best-fitting model represents the best-fitting model of the linear, quadratic, and cubic regression models. (DOCX) [file pone.0179624.s005.docx]

**S3 Table. Results of polynomial regression modeling of the developmental trajectories of the rostrum and genu in chimpanzees with and without Pico’s data.**

|  | Region | Best fitting model | *F* | *R^2^* | sig |
| --- | --- | --- | --- | --- | --- |
| Chimpanzee data sample with Pico data | Rostrum | Cubic | 15.20 | 0.65 | 2.18×10^−5^ |
|  | Genu | Cubic | 66.66 | 0.91 | 1.32×10^−9^ |
|  |  |  |  |  |  |
| Chimpanzee data sample without Pico data | Rostrum | Cubic | 34.08 | 0.81 | 4.70×10^−8^ |
|  | Genu | Cubic | 31.77 | 0.82 | 3.42×10^−7^ |
