# Supplementary material for: Developmental trajectory of the corpus callosum from infancy to the juvenile stage: Comparative MRI between chimpanzees and humans
Source: PLoS One. 2017 Jun 27;12(6):e0179624. doi: 10.1371/journal.pone.0179624 (PMC5487015; doi:10.1371/journal.pone.0179624)
Supplement: S1 File — (DOCX) [file pone.0179624.s006.docx]

**S1 File. Comparison of the developmental trajectories of the rostrum and genu in young chimpanzees with and without Pico’s data.**

One of the longitudinally evaluated young chimpanzees (Pico) in this study had complications from spinal cord compression, and died at the age of two years. Although there was no noticeable abnormality on the brain MRI, areas (mm^2^) of the rostrum and the genu were outside the range of the other three chimpanzees. Therefore, we conducted an additional analysis without Pico’s data to assess the potential impact of including Pico in the results. All statistical analyses were performed in accordance with the same statistical analyses in the main text of this paper.

The rostrum and genu increased nonlinearly during the developmental period in both the rostrum and genu in young chimpanzees, both with and without Pico’s data. (S3 Table). Results in mm^2^ are demonstrated in S1 Fig, and in normalized areas in S2 Fig. The developmental trajectories of the rostrum and the genu were very similar with and without Pico’s data. With Pico’s data, the normalized subdivisions at the end of infancy and the end of the juvenile stage were 64% and 94% in the rostrum, respectively, and 61% and 75% in the genu, respectively (S2 Fig). Without Pico’s data, the normalized subdivisions at the end of infancy and the end of juvenile stage were 63% and 94% in the rostrum, respectively, and 65% and 75% in the genu, respectively (S2 Fig). We found that the results without Pico’s data did not change our conclusion obtained from the full dataset.
