# Supplementary material for: Developmental trajectory of the corpus callosum from infancy to the juvenile stage: Comparative MRI between chimpanzees and humans
Source: PLoS One. 2017 Jun 27;12(6):e0179624. doi: 10.1371/journal.pone.0179624 (PMC5487015; doi:10.1371/journal.pone.0179624)
Supplement: S2 File — (DOCX) [file pone.0179624.s007.docx]

**S2 File. Comparison of the measurements of the chimpanzee CC area acquired with different MRI sequences.**

We measured the midsagittal CC area of a brain sample of an adult chimpanzee, scanned with two different sequences, to investigate the effect of the sequence on the measurement.

The brain sample was perfusion-fixed using 10% paraformaldehyde and scanned with the SPGR and FGRE-IrP sequences, using the same MRI scanner (0.2 Tesla General Electric scanner) that was used in the main experiment. The image analyst (T.S.) manually drew the CC boundary of these two images using the same technique applied to measure the midsagittal CC area in the main study.

The absolute percent difference =

|(CC area from FGRE-IrP – CC area from SPGR)|× 100 / CC area from FGRE-IrP

was calculated to evaluate the reproducibility, and was compared to

the inter-rater difference =

|(1^st^ measurement – 2^nd^ measurement)|× 100 / 1^st^ measurement

The absolute percentage difference was 0.2%. The intra-rater difference of the manual delineation was 1.7%. Therefore, we concluded that the difference in the scan protocol had little effect in the quantification of the CC area.
